# Response Surface–Fuzzy Comprehensive Evaluation-Based Inverse Mix Design and Strength Mechanisms of Waste Rock–Unclassified Tailings Cemented Backfill

**DOI:** 10.3390/ma19194189

**Published:** 2026-09-30

**Authors:** Junhui Yao, Dongyang Liu, Junhui Zhang, Dong Liang, Jinyuan Li, Hui Chen

**Affiliations:** 1 Key Laboratory of Green and Efficient Mining and Ecological Restoration in High-Altitude Arid Regions of Xinjiang, Urumqi 830047, China; rockman@xju.edu.cn (J.Y.); dongyangliu2002@163.com (D.L.); zhangjunhui@xju.edu.cn (J.Z.); 17639764031@163.com (J.L.); 2 School of Geology and Mining Engineering, Xinjiang University, Urumqi 830047, China; 3 China National Gold Engineering Co., Ltd., Mining Branch, Xi’an 710000, China; xju_liangdong@163.com

**Keywords:** waste rock–unclassified tailings cemented backfill, inverse mix design, response surface methodology, fuzzy comprehensive evaluation, strength mechanism

## Abstract

This study developed a coupled response surface methodology–fuzzy comprehensive evaluation (RSM–FCE) approach for the inverse mix design of waste rock–unclassified tailings cemented backfill under a target-strength constraint. The unclassified tailings replacement ratio, aggregate-to-cement ratio, and slurry solids concentration were selected as the key factors. A Box–Behnken design (BBD) was used to establish a response-surface model for 28 d uniaxial compressive strength (UCS). Fuzzy comprehensive evaluation was then used to screen candidate mix-design ranges, while low-field nuclear magnetic resonance (LF-NMR) and scanning electron microscopy (SEM) were used to investigate microstructural features associated with strength development. The main effects on UCS decreased in the order of aggregate-to-cement ratio, slurry solids concentration, and unclassified tailings replacement ratio. The strongest interaction occurred between the aggregate-to-cement ratio and slurry solids concentration. Fine-particle filling, hydration-product infilling, interparticle bonding, reduced free water, and suppressed bleeding jointly refined the pore structure and promoted a continuous bonding network and matrix densification. The coupled RSM–FCE analysis identified candidate ranges of 35–40% for the unclassified tailings replacement ratio, 4–6 for the aggregate-to-cement ratio, and 84–86% for the slurry solids concentration. The measured 28 d UCS values ranged from 5.24 to 9.21 MPa and all met the mine’s target-strength requirement.

## 1. Introduction

Cemented backfilling is an important technology for the utilisation of mine solid waste and controlling ground pressure in mined-out areas. Its engineering performance depends strongly on the rational design of backfill mixture proportions [1,2,3,4]. Mix designs that meet mine-strength requirements are commonly identified through orthogonal experiments, full-factorial experiments, and repeated laboratory trials in which the parameters are adjusted, and the strength is retested [5,6,7]. This process is time-consuming, consumes substantial material, and has low optimisation efficiency. Efficient mix design under a target-strength constraint therefore requires a systematic understanding of factor effects, the underlying strength mechanisms, and an inverse-design method based on these relationships.

The strength of cemented backfill is jointly controlled by several mix-design parameters. Key parameters, including the unclassified tailings replacement ratio, aggregate-to-cement ratio, and slurry solids concentration, affect both slurry transportability and strength development [8]. Previous studies have shown that binder type and dosage, aggregate gradation, solid-waste admixture content, fine-particle content, and chemical admixtures all influence backfill strength [9,10,11,12,13]. Taguchi designs, orthogonal designs, single-factor experiments, and repeated mixture trials have been widely used to characterise these relationships [9,10,11,12,13]. However, increasing the number of factors and their levels substantially increases the number of experimental combinations and the associated workload. Moreover, the main effects and interactions among factors have not been quantified sufficiently, leaving the multivariable strength response of waste rock–unclassified tailings cemented backfill incompletely understood.

Backfill strength is not a simple linear response to mix-design parameters. It emerges from the combined effects of particle packing, pore structure, and hydration-induced bonding. The unclassified tailings replacement ratio regulates the coarse-to-fine aggregate proportion and particle-size distribution, thereby altering the voids within the waste-rock skeleton and the overall packing density. The aggregate-to-cement ratio controls binder dosage and consequently affects hydration-product formation, pore filling, and interparticle bridging. The slurry solids concentration governs free-water content, bleeding, and particle segregation, which influence the development of capillary and connected pores after hardening [14,15,16]. Low-field nuclear magnetic resonance (LF-NMR), scanning electron microscopy (SEM), mercury intrusion porosimetry (MIP), and X-ray diffraction (XRD) have been used to examine these structures. These methods have linked pore refinement, improved particle contact, and a more continuous bonding network to higher macroscopic strength [17,18]. Nevertheless, most studies have focused on individual factors or isolated microstructural features. A unified explanation linking factor main effects and interactions to pore and bonding-structure evolution remains lacking.

Building on an understanding of the multivariable strength response and its underlying microstructural mechanisms, establishing a quantitative relationship between mix-design parameters and target strength is essential for inverse mix design. Response surface methodology (RSM) and the Box–Behnken design (BBD) can establish continuous relationships between mix-design factors and backfill strength with relatively few experiments. They also quantify factor main effects and interactions and have therefore been widely applied to strength prediction and mix optimisation [12,19]. Machine-learning methods can represent complex nonlinear relationships, but they generally require larger datasets and offer limited model interpretability [20,21,22,23]. By contrast, fuzzy comprehensive evaluation (FCE) is well-suited to strength-grade classification and parameter-range screening [24,25]. Existing studies have mainly addressed forward prediction for known mixtures or optimisation within predefined ranges, with limited attention to feasible-range identification under a target-strength constraint. Coupling the continuous predictive capability of RSM with the classification and range-screening capability of FCE may address this limitation. Therefore, the principal methodological gap is that comparatively limited attention has been given to inverse mix-design approaches that translate a prescribed strength requirement into corresponding mix-design parameter ranges and subsequently into specific mixture combinations.

In this study, waste rock–unclassified tailings cemented backfill was investigated, with the unclassified tailings replacement ratio, aggregate-to-cement ratio, and slurry solids concentration selected as the key mix-design factors. The investigated ranges of these factors were 25–40% for the unclassified tailings replacement ratio, 4–10 for the aggregate-to-cement ratio, and 80–86% for the slurry solids concentration. A three-factor, three-level Box–Behnken design comprising 17 runs was conducted, and the 28 d uniaxial compressive strength (UCS) was used as the response. The specimens were cured at 20 °C and 98% relative humidity before strength and microstructural testing. A response-surface model was established for 28 d uniaxial compressive strength (UCS), and the factor main effects and interactions were quantified. Low-field nuclear magnetic resonance (LF-NMR) and scanning electron microscopy (SEM) were used to characterise the pore structure and microscopic bonding morphology under different mix conditions. The mine required a 28 d UCS greater than 5 MPa, which was used as the target-strength constraint for the subsequent inverse-design procedure. A coupled RSM–FCE framework was then developed to screen candidate mix-design ranges, determine specific parameter combinations, and experimentally verify the recommendations under a target-strength constraint. Accordingly, the principal contribution of this study is the development of a target-strength-oriented RSM–FCE inverse-design workflow that links a prescribed UCS requirement with parameter screening, specific mixture selection, and experimental verification, thereby extending the design process beyond conventional forward factor–strength prediction. The findings provide a methodological basis for target-strength-oriented mixture design of waste rock–unclassified tailings cemented backfill.

## 2. Materials and Methods

**Figure 1:**
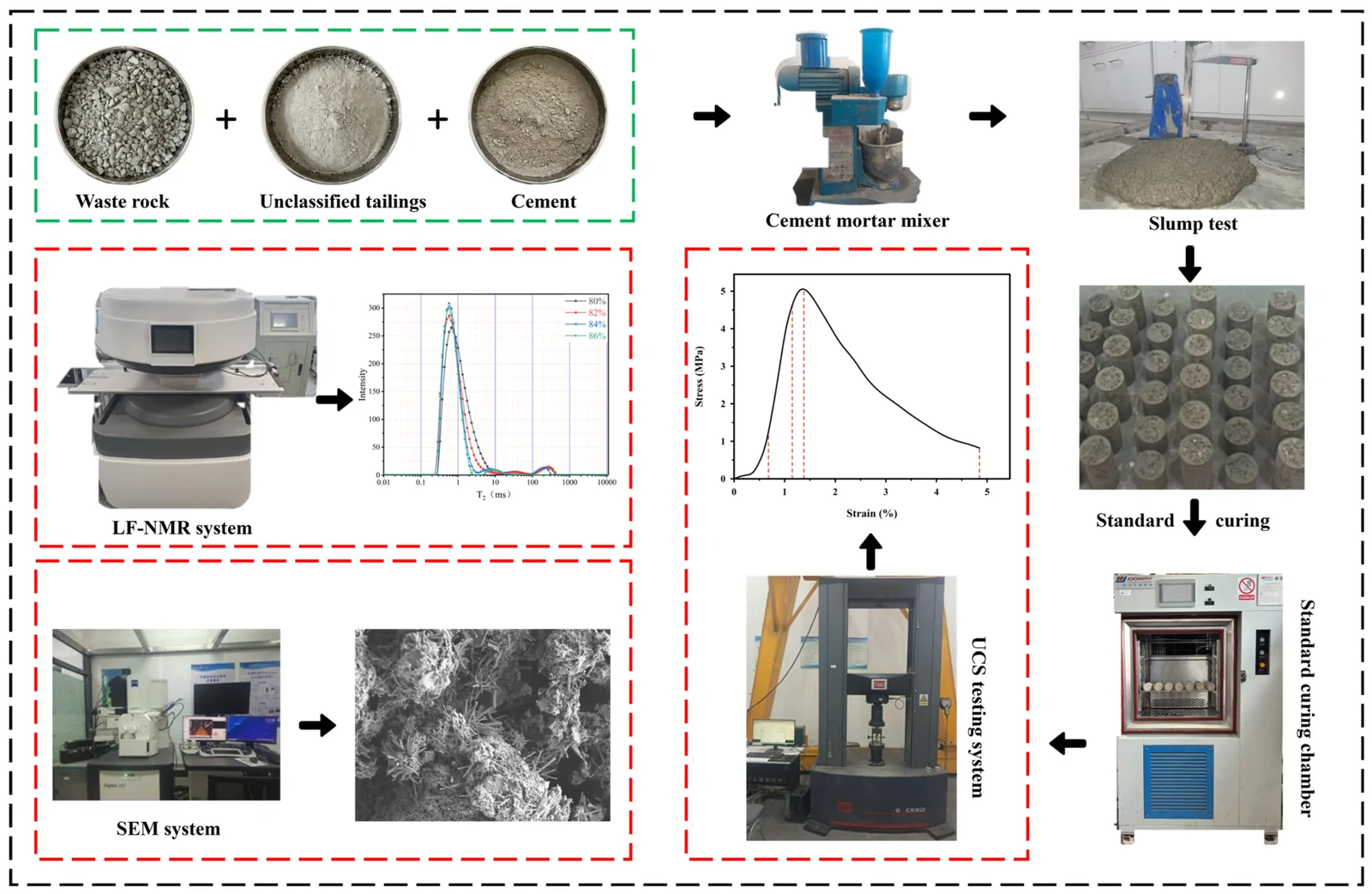
Experimental workflow for backfill preparation and testing.

The overall experimental workflow is shown in Figure 1 and comprised raw-material proportioning, fresh-slurry slump testing, standard specimen curing, UCS testing, and LF-NMR and SEM characterisation.

### 2.1. Raw Materials and Specimen Preparation

**Figure 2:**
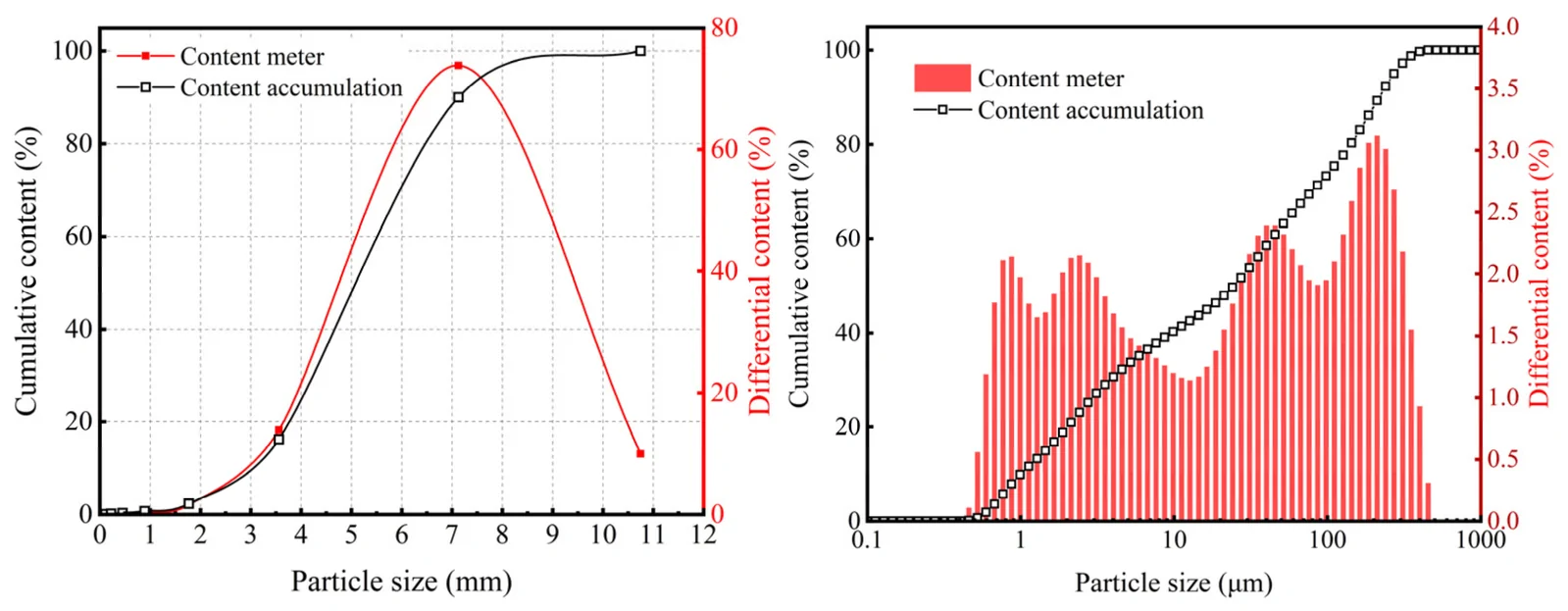
Particle-size distributions of waste rock and unclassified tailings.

The waste rock and unclassified tailings were obtained from the Kalatongke copper–nickel mine in Xinjiang, China. The waste rock had a maximum particle size of 12 mm, and the unclassified tailings were flotation tailings discharged from the mine concentrator. P·O 42.5 ordinary Portland cement was used as the binder. The basic physical properties of the aggregates and the chemical compositions of all raw materials are listed in Table 1 and Table 2, respectively. The apparent densities of the waste rock and unclassified tailings were determined using the wide-mouth bottle and volumetric-flask methods, respectively. Their bulk densities were measured under natural loose-packing conditions using a calibrated cylindrical container, and the packing density was calculated from the ratio of bulk density to apparent density. Porosity was calculated as 100% minus the packing density. The moisture contents were determined by oven drying at 105 ± 5 °C to constant mass. The chemical compositions of the waste rock, unclassified tailings, and cement were determined by X-ray fluorescence (XRF) analysis (Thermo Fisher Scientific, Brno, Czech Republic). The apparent densities of the waste rock and unclassified tailings were 2.74 and 2.68 g/cm^3^, and their moisture contents were 4.97% and 15.24%, respectively. The amount of mixing water was corrected for the as-received moisture contents of the raw materials for each batch. The corrected mixing-water dosage was calculated based on the target slurry solids concentration, considering the water already contained in the raw materials. SiO_2_ and Al_2_O_3_ were the predominant components of both aggregate fractions, while the cement contained 58.05% CaO. As shown in Figure 2, the waste-rock particle size was concentrated between 6 and 8 mm. The unclassified tailings had a median particle size (d_50_) of 24.62 μm and an effective maximum particle size of approximately 454 μm. Particles smaller than 20 and 38 μm accounted for 46.38% and 56.13%, respectively. This pronounced particle-size contrast facilitated the formation of a blended coarse–fine aggregate system.

Waste rock, unclassified tailings, cement, and water were weighed according to the specified proportions. The mixing-water dosage was corrected for the moisture contents of the raw materials to achieve the target slurry solids concentration. After thorough mixing, the fresh slurry was cast in cylindrical moulds measuring 50 mm in diameter and 100 mm in height. The specimens were demoulded after 24 h and cured for 28 d in a standard curing chamber at 20 °C and 98% relative humidity. The specimens were then subjected to UCS and microstructural tests.

### 2.2. Experimental Procedure

The unclassified tailings replacement ratio, aggregate-to-cement ratio, and slurry solids concentration were selected to represent blended-aggregate gradation, binder dosage, and slurry water content, respectively. Their ranges were determined from preliminary aggregate packing/gradation tests, actual mine-filling practice, and preliminary slump tests, respectively. The unclassified tailings replacement ratio, aggregate-to-cement ratio, and slurry solids concentration were set to 25–40%, 4–10, and 80–86%, respectively. The 25–40% replacement range maintained the waste-rock coarse-aggregate skeleton while providing relatively high packing density and suitable gradation; the aggregate-to-cement ratio of 4–10 reflected actual mine-filling practice; and the slurry solids concentration of 80–86% avoided excessively dry slurry at high concentrations and segregation at low concentrations. Other variables, such as binder type, curing conditions, and aggregate characteristics, were kept constant to maintain a consistent material system and ensure a focused evaluation of the selected mixture-design factors. Each factor was tested at low, intermediate, and high levels. The lower bound, midpoint, and upper bound of each range were used as the three BBD levels (Table 3).

A three-factor, three-level BBD comprising 17 runs was generated using Design-Expert 13, with coded levels of −1, 0, and +1 [26]. The design included five replicated centre-point runs, which provided the pure-error estimate for the subsequent analysis of variance (ANOVA). The mean 28 d UCS values were fitted to a second-order polynomial response-surface model containing linear, two-factor interaction, and quadratic terms according to standard RSM procedures [27], and the model was evaluated by ANOVA.

### 2.3. Test Methods

The slump of the freshly mixed slurry was measured using a standard slump cone placed on a level base plate. The thoroughly mixed slurry was placed and compacted in the cone, which was then lifted vertically. After deformation ceased, the vertical difference between the cone height and the highest point of the slurry was recorded as the slump.

The UCS was measured after 28 d of curing using an MTS 815 rock-mechanics testing system (MTS Systems Corporation, Eden Prairie, MN, USA). Displacement-controlled loading was applied at 0.5 mm/min. For each BBD run, three replicate specimens were tested for 28 d UCS, and the mean and standard deviation of the three measurements were calculated. The mean UCS was used as the response value for RSM fitting.

For LF-NMR testing, the specimens were cured for 28 d, cooled to room temperature, and water-saturated for 24 h. Surface water was blotted off, and the transverse relaxation-time (*T*_2_) distribution was measured immediately. The measurements were performed using an Hxcore magnetic resonance online analysis and imaging system (Suzhou Niumag Analytical Instrument Co., Ltd., Suzhou, China) with a proton resonance frequency of 12.061503 MHz (corresponding to a magnetic field strength of approximately 0.283 T). The transverse relaxation signals were acquired using a Carr–Purcell–Meiboom–Gill (CPMG) sequence with an echo time (TE) of 0.12 ms and 50,000 echoes.

For SEM analysis, representative fragments were collected from the interior of each specimen and sputter-coated with gold. Hydration products, pores, microcracks, and particle interfaces were examined using a ZEISS SIGMA 300 field-emission scanning electron microscope (Carl Zeiss Microscopy GmbH, Jena, Germany).

## 3. Effects and Mechanisms of Mix-Design Factors

### 3.1. Response-Surface Model

Based on the model-fitting procedure described in Section 2.2, the fitted second-order polynomial model for the 28 d UCS is expressed in standardised coded variables as follows [27]:

(1) $$\begin{array}{c} y=0.3425x_{1}-3.3x_{2}+1.81x_{3}-0.1x_{1}x_{2}-0.28x_{1}x_{3}\\ -1.22x_{2}x_{3}-0.0373x_{1}^{2}+1.6x_{2}^{2}+0.7653x_{3}^{2}+2.81 \end{array}$$

In Equation (1), y is the 28 d UCS of the backfill specimen (MPa), while x_1_, x_2_, and x_3_ are the standardised unclassified tailings replacement ratio, aggregate-to-cement ratio, and slurry solids concentration, respectively.

The response-surface results are listed in Table 4. UCS increased as the aggregate-to-cement ratio decreased, the slurry solids concentration increased, and the unclassified tailings replacement ratio increased moderately. The greatest increase occurred at a low aggregate-to-cement ratio and a high slurry solids concentration.

The statistical significance and adequacy of the quadratic response-surface model were evaluated by ANOVA, as summarised in Table 5.

The overall quadratic model was highly significant (F = 131.19, *p* < 0.0001), whereas the lack of fit was not significant (F = 3.83, *p* = 0.1136). The model yielded an *R*^2^ of 0.9941, an adjusted *R*^2^ of 0.9865, and a predicted *R*^2^ of 0.9277. The standard deviation was 0.3376 MPa, the coefficient of variation was 8.64%, the predicted residual error sum of squares (PRESS) was 9.79, and the Adeq Precision value was 41.2535. These statistical indicators indicate satisfactory goodness of fit and predictive performance of the model within the investigated design space.

**Figure 3:**
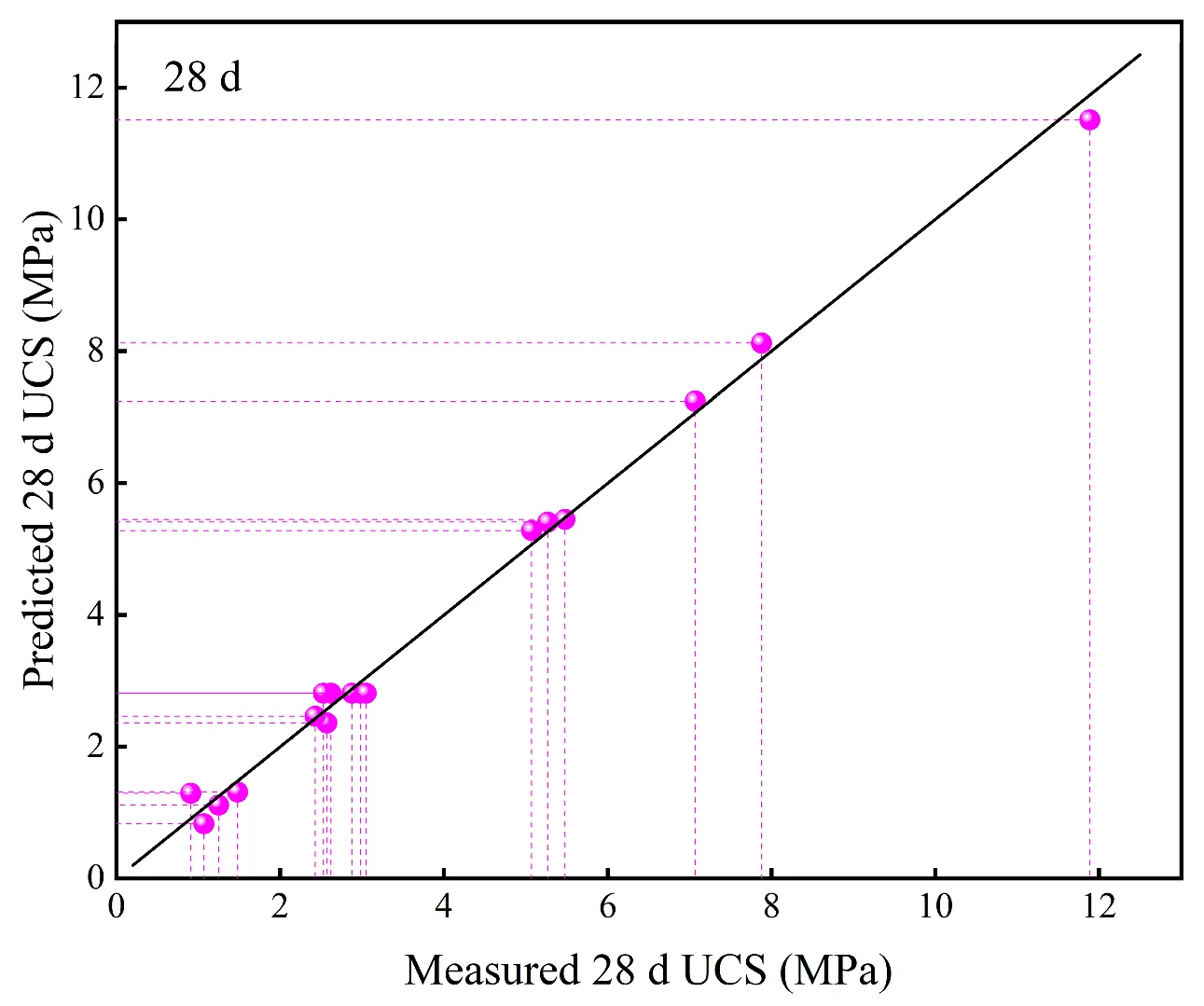
Predicted versus measured 28 d UCS.

The predicted and measured 28 d UCS values lay close to the 1:1 reference line (Figure 3), further indicating good agreement between the model predictions and experimental measurements.

### 3.2. Effects of Mix-Design Factors

The main effects of the three factors were compared using the magnitudes and significance of the linear coefficients in the standardised regression equation. Within the experimental domain, their effects on 28 d UCS decreased in the following order: aggregate-to-cement ratio x_2_ > slurry solids concentration x_3_ > unclassified tailings replacement ratio x_1_.

**Figure 4:**
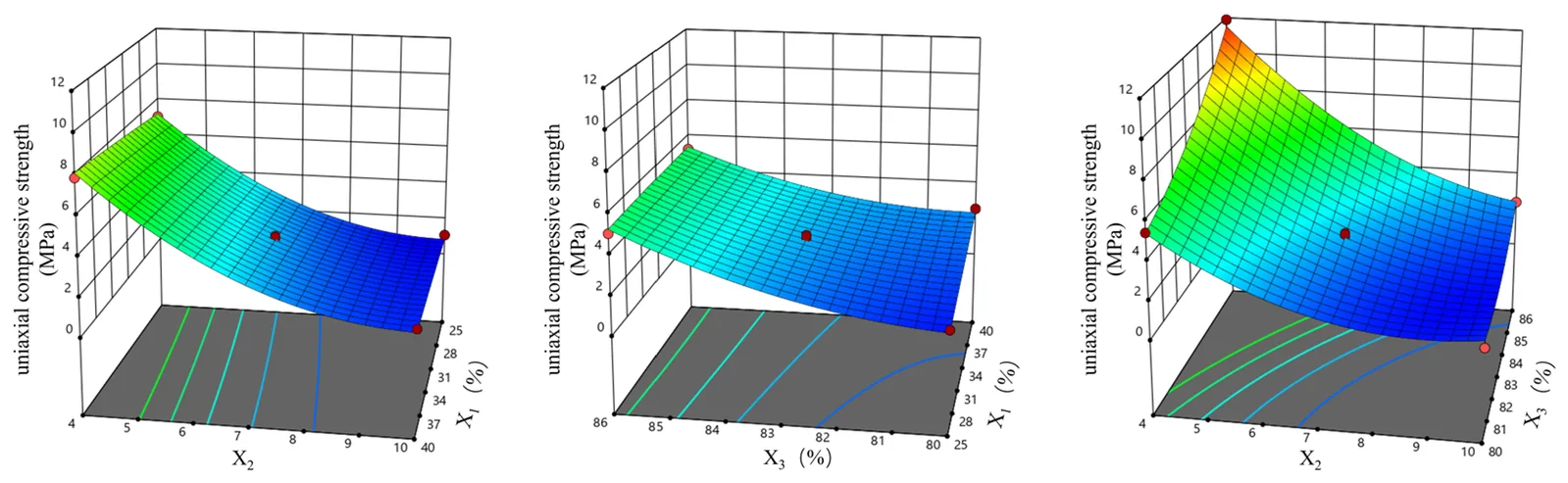
Interaction response surfaces for 28 d UCS.

To further investigate the pairwise interactions among the mix-design factors and their effects on backfill strength, three-dimensional response surfaces were generated for the unclassified tailings replacement ratio–aggregate-to-cement ratio, unclassified tailings replacement ratio–slurry solids concentration, and aggregate-to-cement ratio–slurry solids concentration interactions, as shown in Figure 4.

As shown in Figure 4, the 28 d UCS generally increased with a decrease in the aggregate-to-cement ratio, an increase in the slurry solids concentration, and a moderate increase in the unclassified tailings replacement ratio. However, the response surfaces differed in curvature and in the magnitude of strength increase under different factor combinations, indicating that backfill strength was jointly governed by multiple mix-design factors rather than linearly controlled by any single factor.

Among the three pairwise interactions, the response surface corresponding to the aggregate-to-cement ratio and slurry solids concentration exhibited the greatest curvature and the steepest strength gradient, indicating a strong interaction between the two factors. When the aggregate-to-cement ratio was low, increasing the slurry solids concentration produced a more pronounced strength-enhancing effect. In contrast, when the aggregate-to-cement ratio was high, the increase in strength remained limited even as the slurry solids concentration increased. These results indicate that the strength-enhancing effect of slurry solids concentration was constrained by the aggregate-to-cement ratio, and that the interaction between these two factors played an important role in the backfill strength response.

In contrast, the interactions of the unclassified tailings replacement ratio with the aggregate-to-cement ratio and slurry solids concentration were relatively weak, as reflected by the comparatively flat response surfaces. A moderate increase in the unclassified tailings replacement ratio could improve the blended-aggregate gradation to some extent; however, its strength-enhancing effect remained jointly constrained by the levels of the other two factors.

### 3.3. Pore Structure and Microstructural Morphology

LF-NMR was first used to characterise the pore-structure variations in the 28 d specimens and to identify microstructural features associated with the strength differences among mixtures. Under identical test conditions and assuming comparable surface relaxivity, the transverse relaxation time (T_2_) is generally positively correlated with pore size. Therefore, the peak position, peak intensity, and distribution width of the T_2_ spectra were used to characterise relative differences in pore structure among mixtures. It should be noted that T_2_ relaxation is influenced not only by pore size but also by surface relaxivity, pore geometry, saturation state, and magnetic-field conditions. Therefore, the T_2_ distributions were used to characterise relative pore-structure evolution under the given testing conditions rather than to obtain absolute pore-size values.

**Figure 5:**
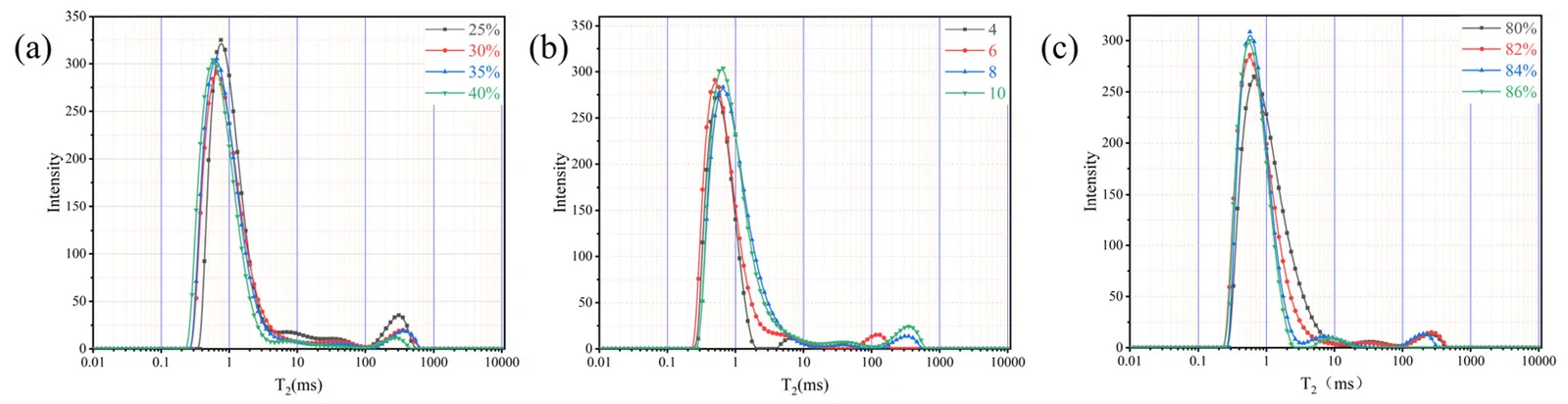
T_2_ relaxation spectra illustrating pore-structure characteristics of 28 d backfill specimens: (**a**) different unclassified tailings replacement ratios; (**b**) different aggregate-to-cement ratios; (**c**) different slurry solids concentrations.

All (T_2_) distributions were dominated by a short-relaxation-time peak, with only weak secondary peaks or tails in the long-relaxation region (Figure 5). This pattern indicated that small pores predominated, with only a small fraction of large or connected pores. Increasing the unclassified tailings replacement ratio from 25% to 40% lowered and shifted the principal peak to the left and narrowed the distribution. Increasing the aggregate-to-cement ratio from 4 to 10 increased and shifted the principal peak to the right and broadened the distribution. Increasing the slurry solids concentration from 80% to 86% shifted the peak to the left and reduced both the long-relaxation signal and the distribution width. Thus, the observed shifts in the T_2_ distributions indicate a tendency toward a refined pore structure and a narrower T_2_ distribution with increasing unclassified tailings replacement ratio and slurry solids concentration, whereas increasing the aggregate-to-cement ratio exhibited a tendency toward a broader T_2_ distribution and pore coarsening.

SEM was subsequently used to examine particle contacts, pore defects, and the distribution of hydration products in specimens prepared with different mixture proportions after 28 d of curing.

**Figure 6:**
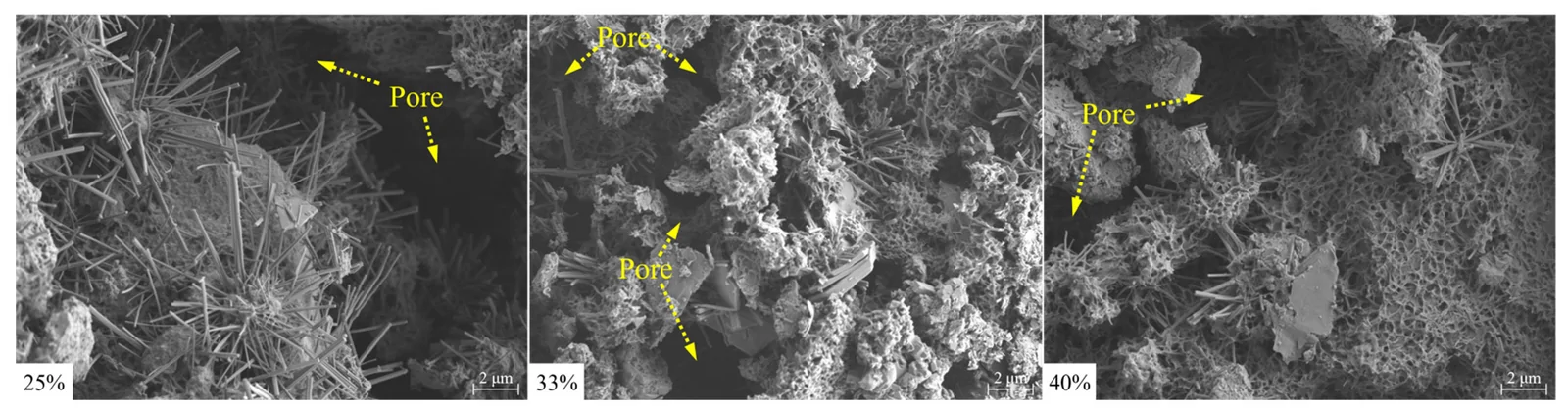
SEM micrographs of 28 d specimens at different unclassified tailings replacement ratios.

**Figure 7:**
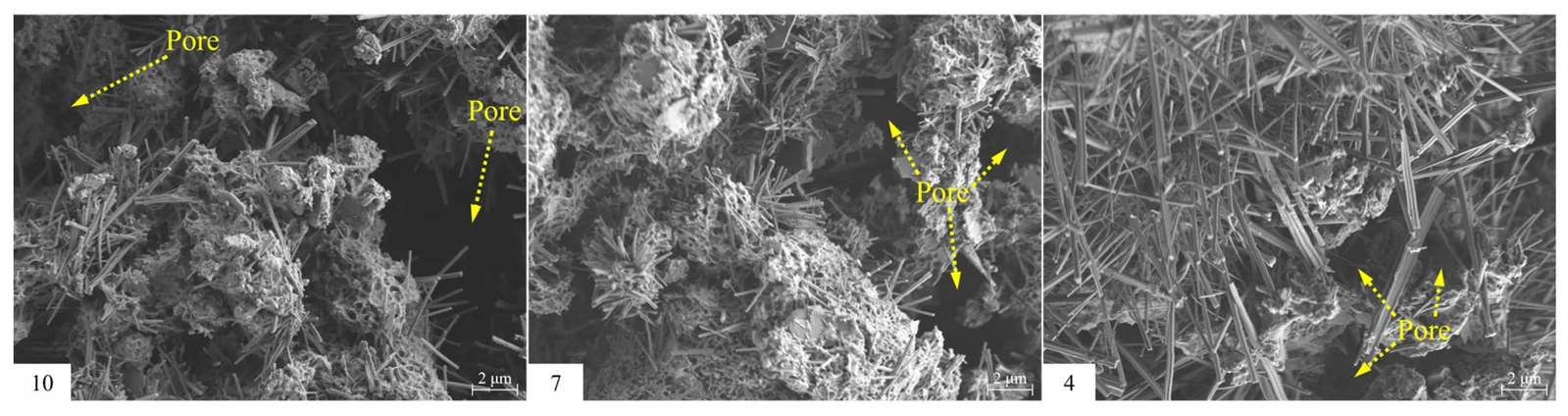
SEM micrographs of 28 d specimens at different aggregate-to-cement ratios.

**Figure 8:**
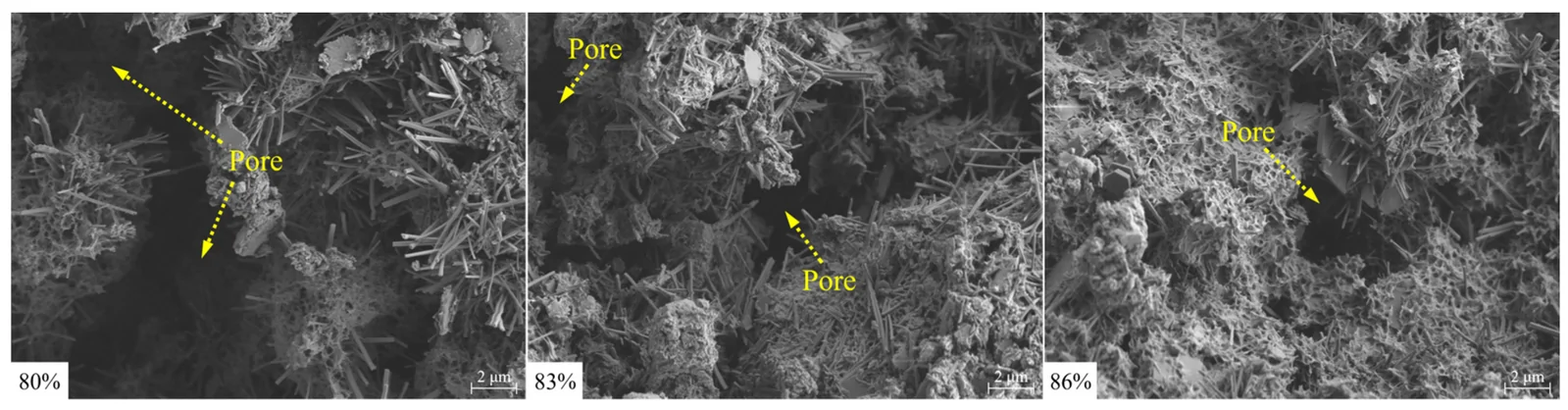
SEM micrographs of 28 d specimens at different slurry solids concentrations.

The pore defects, particle-contact states, and hydration-product morphologies differed substantially among the mixtures (Figure 6, Figure 7 and Figure 8). As the unclassified tailings replacement ratio increased, SEM observations showed fewer irregular pores and loose regions, closer particle contacts, and a greater presence of hydration-product-like phases on particle surfaces and between adjacent particles. Decreasing the aggregate-to-cement ratio reduced the number of pores and microcracks and increased the abundance of gel-like phases and needle-shaped crystals on and between particles. The bonding structure changed from a dispersed arrangement to a more continuous network. Based on their characteristic morphologies, these phases were tentatively attributed to calcium silicate hydrate (C–S–H)-like gel and ettringite (AFt)-like needle-shaped phases. Increasing the slurry solids concentration further reduced pores, local defects, and loose regions. It also improved microstructural uniformity, compactness, and continuity and strengthened the bonding between aggregate particles and hydration products.

**Figure 9:**
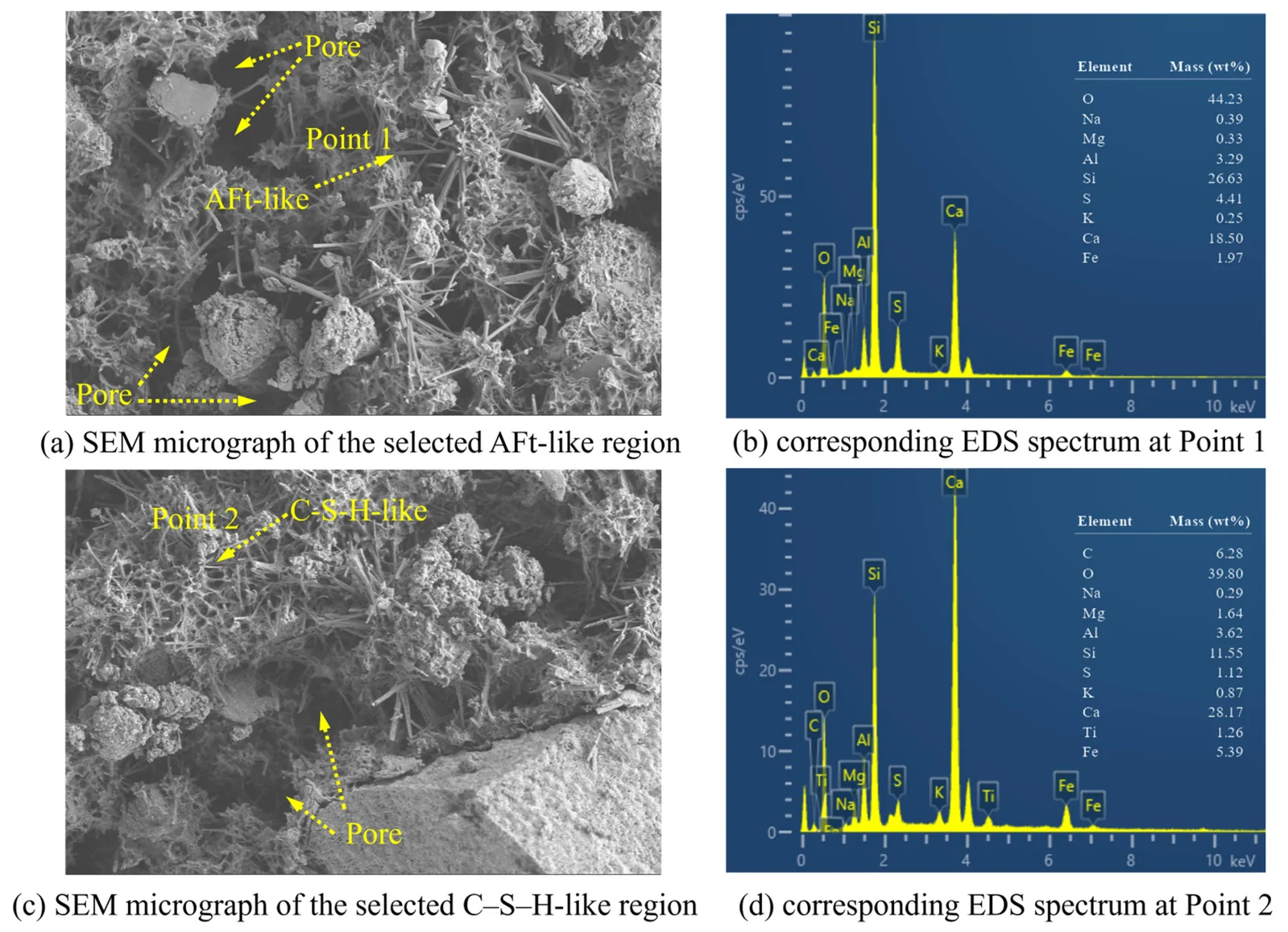
SEM–EDS analysis of representative hydration-product-like regions in 28 d backfill specimens.

To further characterise the elemental compositions of these hydration-product-like regions, representative areas with gel-like and needle-shaped morphologies were selected for SEM–EDS analysis (Figure 9). The EDS results showed that the needle-shaped region contained Ca, Al, and S elements, which were consistent with the elemental characteristics commonly associated with AFt-like phases. The gel-like region mainly exhibited Ca and Si signals, which were consistent with the elemental characteristics commonly associated with C–S–H-like phases. The elemental compositions obtained from EDS analysis agreed with the morphological features observed in SEM micrographs.

### 3.4. Mechanisms of Factor Effects

**Figure 10:**
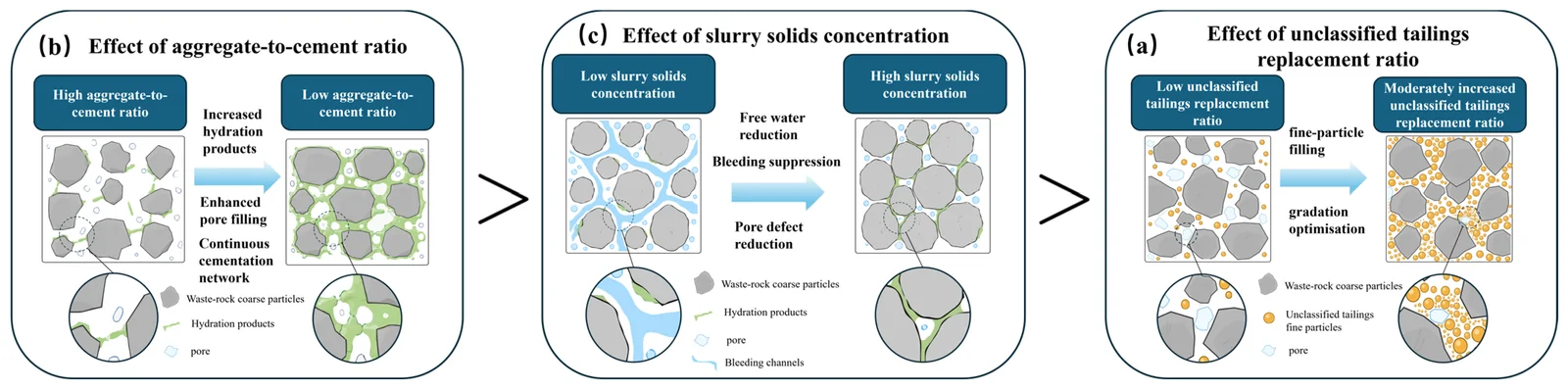
Microstructural mechanisms of mix-design factor effects on backfill strength: (**a**) different unclassified tailings replacement ratios; (**b**) different aggregate-to-cement ratios; (**c**) different slurry solids concentrations.

The effects of the mix-design factors were interpreted from the following three perspectives: blended-aggregate gradation, binder availability, and the free-water state of the system. The interpretation combined the strength responses in Section 3.2 with the microstructural evidence in Section 3.3 and is summarised schematically in Figure 10.

The unclassified tailings replacement ratio primarily influenced strength variation through its effects on the coarse-to-fine particle proportion and blended-aggregate gradation. A moderate increase was associated with increased filling of waste-rock skeleton voids by fine particles, improved blended-aggregate gradation, and a tendency toward microstructural densification with refined pore features [28]. These changes also may facilitate pore filling and bonding by hydration products, which could contribute to increased matrix densification, structural compactness, and strength [29].

The aggregate-to-cement ratio primarily controlled the binder dosage per unit mass of aggregate. Decreasing this ratio increased binder availability and consequently was associated with more evident hydration-product-like features, pore filling, aggregate-surface coverage, and a more continuous bonding morphology [30]. These observations may help explain its strong main effect on UCS.

The slurry solids concentration primarily affected strength by regulating free-water content, bleeding, and segregation. Within the experimental range, increasing this concentration reduced excess free water, suppressed bleed channels and particle segregation, and lowered the likelihood of capillary and connected pore formation. It also produced a more uniform particle distribution and bonding structure, which was associated with matrix densification and strength development [30].

**Figure 11:**
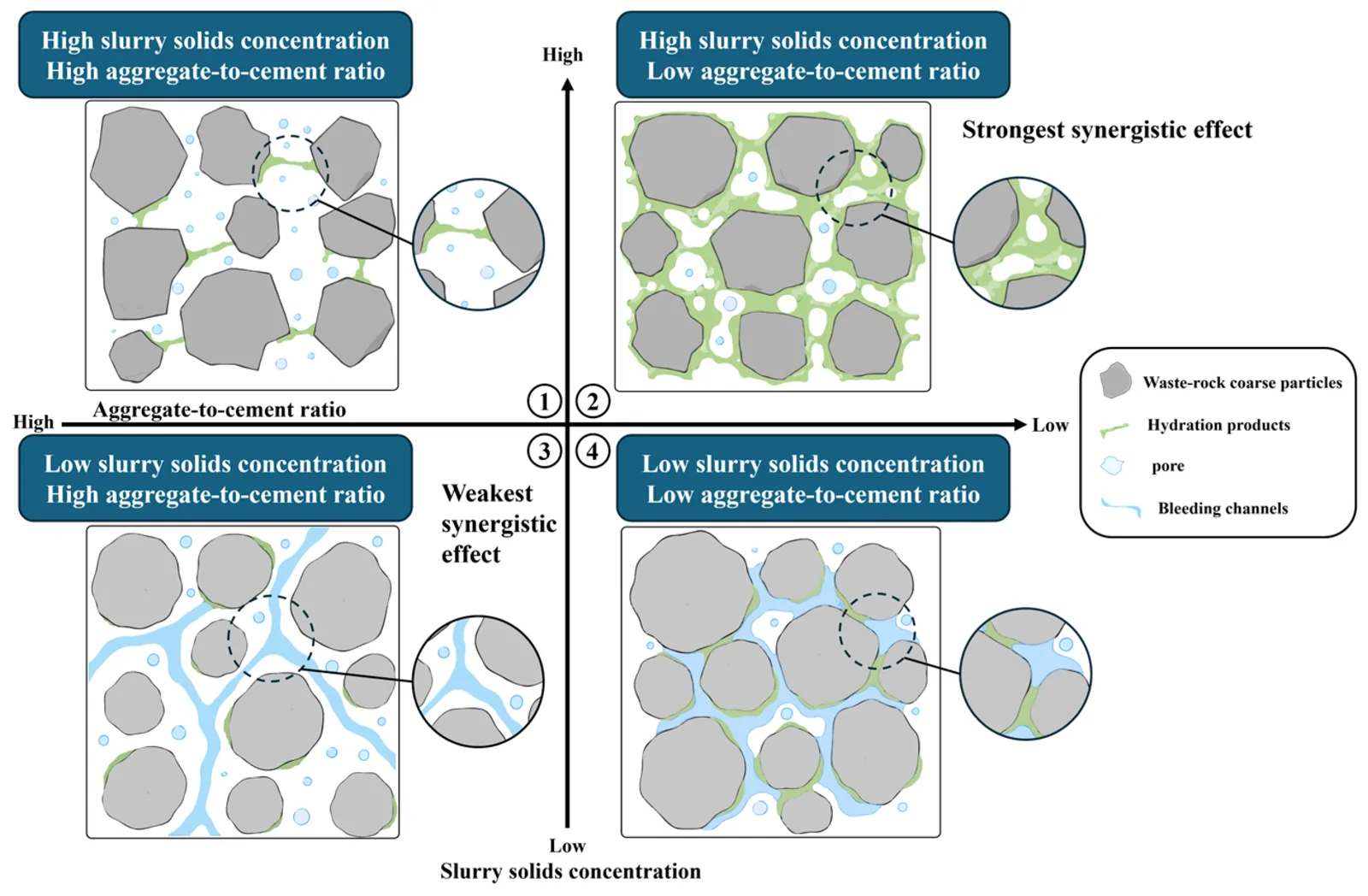
Mechanism of the aggregate-to-cement ratio–slurry solids concentration interaction.

**Figure 12:**
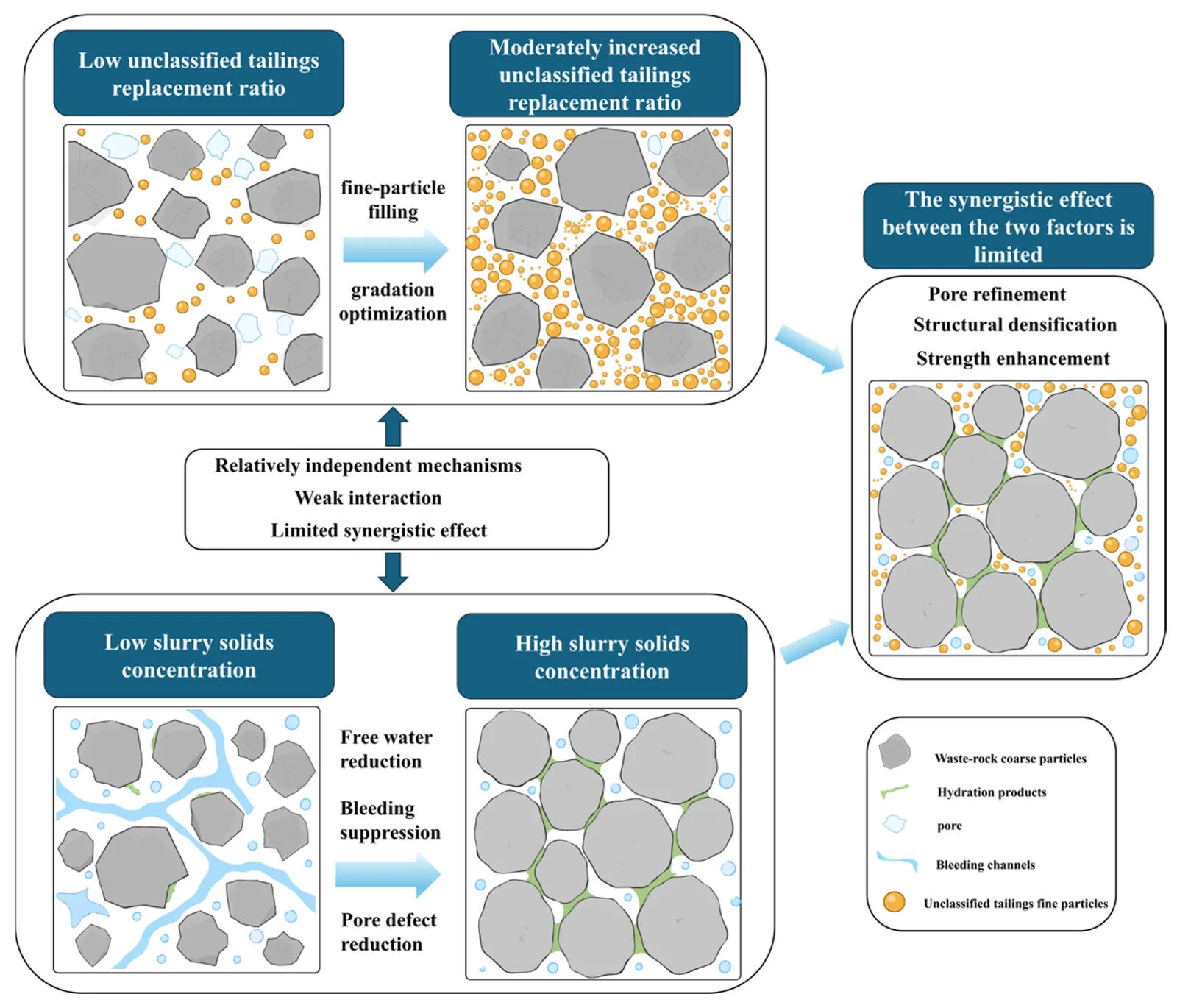
Mechanism of the unclassified tailings replacement ratio–slurry solids concentration interaction.

These results indicate that the three mix-design factors regulate the pore and bonding structures through aggregate gradation, binder availability, and water state, respectively. When two factors change simultaneously, these pathways may reinforce or constrain one another, producing interactions of different magnitudes. The possible mechanisms underlying the aggregate-to-cement ratio–slurry solids concentration interaction and the unclassified tailings replacement ratio–slurry solids concentration interaction are discussed from this perspective in Figure 11 and Figure 12.

The strong interaction between the aggregate-to-cement ratio and slurry solids concentration likely arose from the joint regulation of binder availability and water state (Figure 11). At a low aggregate-to-cement ratio and a high slurry solids concentration, sufficient binder and relatively little free water favoured both hydration-product formation and effective pore filling. These conditions promoted a continuous bonding network, reduced pore defects, and increased structural compactness and strength [30,31]. Conversely, a high aggregate-to-cement ratio or low slurry solids concentration produced insufficient binder or excessive free water. Hydration-product infilling and particle bonding were consequently restricted, while local pores and bleed channels formed more readily, limiting strength development [32].

Figure 12 further illustrates the weaker interaction between the unclassified tailings replacement ratio and slurry solids concentration. The former contributed to matrix densification primarily through fine-particle filling and gradation optimisation, whereas the latter promoted densification mainly by reducing free water and suppressing bleed channels. Because these pathways were relatively independent, their synergistic effect was limited. Their response-surface interaction was therefore weaker than that between the aggregate-to-cement ratio and slurry solids concentration.

Overall, the LF-NMR and SEM results provided qualitative microstructural evidence supporting the observed factor effects through variations in pore structure, particle contacts, and bonding morphology. The factor sensitivities derived from the response-surface model were subsequently used to determine the FCE weights for candidate-range screening and inverse mix design under a target-strength constraint.

## 4. RSM–FCE-Based Inverse Mix Design

### 4.1. Coupled Inverse-Design Framework

Previous RSM studies have mainly focused on forward prediction of backfill strength and identification of a single optimum mixture [33]. Feasible mix-design ranges under a target-strength constraint have received less attention. A coupled RSM–FCE inverse-design framework was therefore developed. First, factor sensitivities from the RSM model were used to determine the FCE weights, and strength-grade classification was used to screen candidate parameter ranges. The RSM regression equation was then used to identify recommended mixtures within these ranges, which were verified experimentally. By combining continuous RSM prediction with FCE-based range screening, the framework enabled stepwise inversion from a target strength to candidate ranges and specific parameter combinations.

### 4.2. FCE Model and Factor Weights

The unclassified tailings replacement ratio, aggregate-to-cement ratio, and slurry solids concentration were used as the evaluation factors in the FCE model. The factor set was defined as follows:

U = {u_1_, u_2_, u_3_}

In this set, u_1_, u_2_, and u_3_ denote the unclassified tailings replacement ratio, aggregate-to-cement ratio, and slurry solids concentration, respectively. The evaluation set for strength-grade classification was defined as follows:

V = {v_1_, v_2_, v_3_}

Here, v_1_, v_2_, and v_3_ denote strength grades 1, 2, and 3, respectively. The grades were ordered from low to high strength, with grade 1 representing the lowest strength range and grade 3 the highest.

Dedicated mixture experiments were used to establish the relationship between strength grades and corresponding factor ranges based on the measured UCS values. The resulting grades provided a strength-based evaluation framework for subsequent FCE analysis and candidate-range screening. The experimental scheme and results used for classification are presented in Table 6, and the resulting strength grades and factor ranges are given in Table 7.

Table 7 shows that the unclassified tailings replacement ratio and slurry solids concentration increased with strength grade. The aggregate-to-cement ratio was an inverse indicator because a lower ratio corresponded to a higher binder dosage and strength grade. This inverse relationship was therefore incorporated when constructing the membership functions.

Trapezoidal membership functions were constructed from the grade boundaries in Table 7 [34]. Separate functions were constructed to describe the contribution of each mix-design factor to different strength grades. The functions for the unclassified tailings replacement ratio *u*_1_, aggregate-to-cement ratio *u*_2_, and slurry solids concentration *u*_3_ were denoted by *μ*_1*j*_(*u*_1_), *μ*_2*j*_(*u*_2_), and *μ*_3*j*_(*u*_3_), respectively, where (j = 1, 2, 3). The functions are given in Equations (2)–(10). The factor membership functions represent grade-related parameter characteristics rather than independent effects of individual factors, while the factor interactions were considered through the response-surface model.

(2) $$\mu_{11}\left(u_{1}\right)=\left\{\begin{array}{ll} 1, & u_{1}\leq 30,\\ \frac{35-u_{1}}{5}, & 30<u_{1}<35,\\ 0, & u_{1}\geq 35. \end{array}\right.$$

(3) $$\mu_{12}\left(u_{1}\right)=\left\{\begin{array}{cccc} 0, & u_{1}\leq 25\;\mathrm{or}{\;u}_{1}\geq 40, & & \\ \frac{u_{1}-25}{5}, & 25<u_{1}<30, & & \\ 1, & 30\leq u_{1}\leq 35, & & \\ \frac{40-u_{1}}{5}, & 35<u_{1}<40. & & \end{array}\right.$$

(4) $$\mu_{13}\left(u_{1}\right)=\left\{\begin{array}{ll} 0, & u_{1}\leq 30,\\ \frac{u_{1}-30}{5}, & 30<u_{1}<35,\;\\ 1, & u_{1}\geq 35. \end{array}\right.$$

(5) $$\mu_{21}\left(u_{2}\right)=\left\{\begin{array}{ll} 1, & u_{2}\geq 8,\\ \frac{u_{2}-6}{2}, & 6<u_{2}<8,\\ 0, & u_{2}\leq 6. \end{array}\right.$$

(6) $$\mu_{22}\left(u_{2}\right)=\left\{\begin{array}{ll} 0, & u_{2}\leq 4\;\mathrm{or}\;u_{2}\geq 10,\\ \frac{u_{2}-4}{2}, & 4<u_{2}<6,\\ 1, & 6\leq u_{2}\leq 8,\\ \frac{10-u_{2}}{2}, & 8<u_{2}<10. \end{array}\right.$$

(7) $$\mu_{23}\left(u_{2}\right)=\left\{\begin{array}{ll} 1, & u_{2}\leq 6,\\ \frac{8-u_{2}}{2}, & 6<u_{2}<8,\\ 0, & u_{2}\geq 8. \end{array}\right.$$

(8) $$\mu_{31}\left(u_{3}\right)=\left\{\begin{array}{ll} 1, & u_{3}\leq 82,\\ \frac{84-u_{3}}{2}, & 82<u_{3}<84,\\ 0, & u_{3}\geq 84. \end{array}\right.$$

(9) $$\mu_{32}\left(u_{3}\right)=\left\{\begin{array}{ll} 0, & u_{3}\leq 80\;\mathrm{or}{\;u}_{3}\geq 86,\\ \frac{u_{3}-80}{2}, & 80<u_{3}<82,\\ 1, & 82\leq u_{3}\leq 84,\\ \frac{86-u_{3}}{2}, & 84<u_{3}<86. \end{array}\right.$$

(10) $$\mu_{33}\left(u_{3}\right)=\left\{\begin{array}{ll} 0, & u_{3}\leq 82,\\ \frac{u_{3}-82}{2}, & 82<u_{3}<84,\\ 1, & u_{3}\geq 84. \end{array}\right.$$

After defining the factor set, evaluation set, and membership functions, the factor weights were determined. To improve objectivity, the FCE weights were derived from the absolute values of the linear main-effect coefficients of the standardised RSM model, which represent the relative contributions of individual factors to UCS variation. The interaction and quadratic terms were retained in the RSM model to describe factor coupling and nonlinear responses, whereas the linear coefficients were selected for FCE weighting to represent the individual contribution of each controllable factor to UCS variation. These coefficients for the unclassified tailings replacement ratio, aggregate-to-cement ratio, and slurry solids concentration were selected as the sensitivity indices and normalised as follows:

$$w_{i}=\frac{\left|\beta_{i}\right|}{\sum_{i=1}^{3}|\beta_{i}|}$$

Here, w_i_ is the weight of the *i*th factor, and β_i_ is its main-effect coefficient in the standardised response-surface model for 28 d UCS. The interaction and quadratic terms describe response-surface curvature and multivariable coupling and were retained in the RSM model used for subsequent inverse design. Because the FCE factor set contained only the three independent mix-design variables, the weights were based on the relative contributions of their main effects to the 28 d UCS response.

The resulting weight vector for the 28 d strength evaluation was as follows:

*W*_28*d*_ = [0.063, 0.605, 0.332]

The weight components correspond, in order, to the unclassified tailings replacement ratio, aggregate-to-cement ratio, and slurry solids concentration. For the 28 d strength evaluation, the aggregate-to-cement ratio had the highest weight, followed by the slurry solids concentration and unclassified tailings replacement ratio. This order agreed with the main-effect ranking obtained from the response-surface model in Section 3.

### 4.3. Strength Classification and Candidate-Range Screening

After the factor set, evaluation set, membership functions, and weight vector had been defined, a fuzzy relation matrix was constructed for strength-grade evaluation. For any mixture, the membership of each factor in each strength grade was calculated using Equations (2)–(10). The fuzzy relation matrix was expressed as follows:

$$R=\left[\begin{array}{ccc} r_{11} & r_{12} & r_{13}\\ r_{21} & r_{22} & r_{23}\\ r_{31} & r_{32} & r_{33} \end{array}\right],0\leq r_{\mathrm{ij}}\leq 1$$

Here, r_ij_ denotes the membership of the *i*th factor in the *j*th strength grade. The fuzzy comprehensive evaluation vector was obtained by multiplying the weight vector *W* by the fuzzy relation matrix *R*:

*B* = *W* ⋅ *R* = (*B*_1_, *B*_2_, *B*_3_)

Here, B_j_ is the overall membership of the mixture in the *j*th strength grade. The weight vector W_28d_ was used for the 28 d strength evaluation. According to the maximum-membership principle, a mixture was assigned to strength grade *j* when B_j_ was the largest component of evaluation vector B [35].

**Figure 13:**
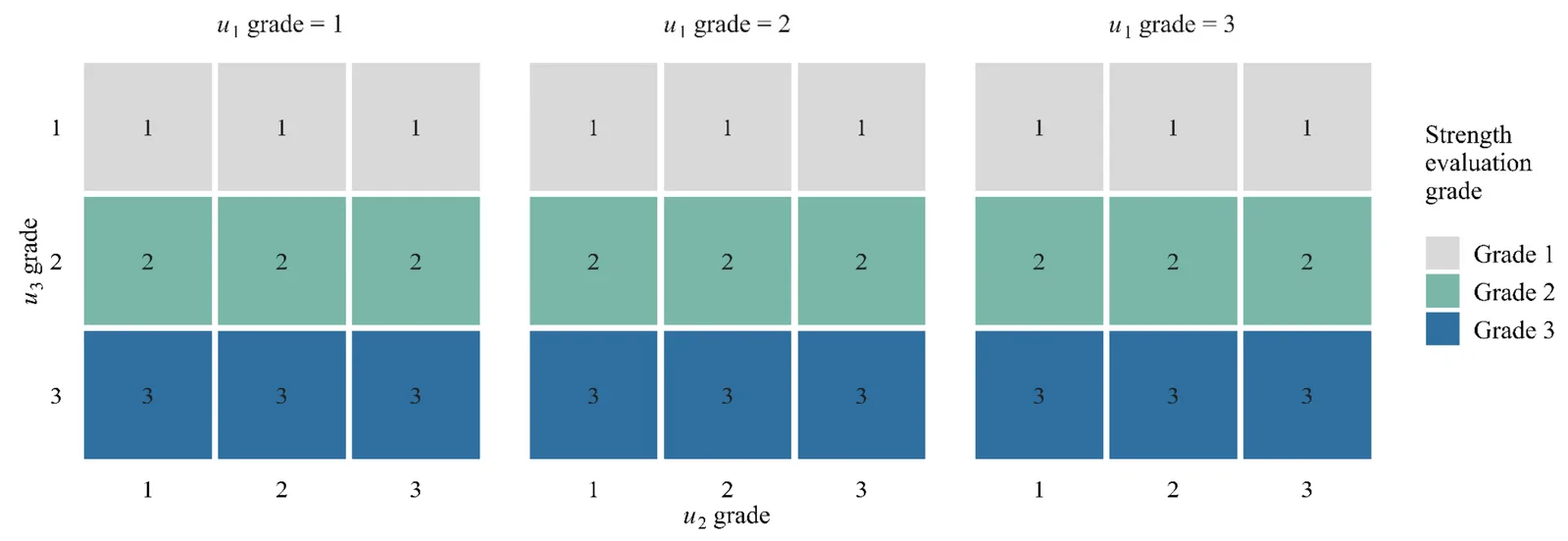
FCE-based strength-grade classification of backfill mixtures. *u*_1_, *u*_2_, and *u*_3_ denote the unclassified tailings replacement ratio, aggregate-to-cement ratio, and slurry solids concentration, respectively. Grades 1, 2, and 3 correspond to the grade-specific factor ranges defined in Table 7.

The consistency between the FCE classification results and the experimental strength grades was evaluated using the 17 response-surface mixtures. The evaluation was conducted based on the mixtures included in the response-surface design, and the results were used to examine the consistency between the FCE classification and the experimentally obtained strength grades. A membership matrix was calculated from the parameters of each mixture, and the 28 d strength-grade evaluation was obtained using the corresponding weight vector (Figure 13).

The FCE and measured strength grades agreed for 14 of the 17 mixtures, corresponding to a classification agreement of 82.35%. The three inconsistent classifications occurred near the boundaries between strength grades, reflecting the uncertainty associated with converting continuous UCS values into discrete evaluation levels. The response-surface-weighted FCE model therefore distinguished the 28 d strength grades of the mixtures with reasonable reliability. The agreement reflects the consistency between the FCE-derived strength classifications and the experimentally measured strength grades based on categorical evaluation.

The copper–nickel mine required a 28 d UCS greater than 5 MPa. In Table 7, grade 3 spanned 5.274–12.250 MPa, with a lower bound slightly above the target. Grade 3 was therefore adopted as a conservative screening criterion, and its factor ranges were treated as candidate ranges for subsequent RSM-based inverse design. The resulting ranges were 35–40% for the unclassified tailings replacement ratio, 4–6 for the aggregate-to-cement ratio, and 84–86% for the slurry solids concentration. These FCE-derived ranges were used as conservative candidate screening intervals to narrow the original design space for subsequent RSM-based inverse design. Because the classification provides a screening rather than an exhaustive delineation of the target-strength domain, mixtures outside these intervals may also satisfy the 28 d UCS requirement (>5 MPa). FCE thus provided the initial strength-grade classification and range screening, while specific recommended mixtures were subsequently identified using the response-surface regression equation.

### 4.4. Inverse Design and Experimental Verification

Section 4.3 identified FCE-screened candidate factor ranges under the target constraint of a 28 d UCS greater than 5 MPa. These ranges were 35–40% for the unclassified tailings replacement ratio, 4–6 for the aggregate-to-cement ratio, and 84–86% for the slurry solids concentration. The unclassified tailings replacement ratio was selected as 40% within the FCE-screened candidate range of 35–40%, considering the engineering objective of maximising tailings utilisation while satisfying the mechanical performance requirement. This upper-bound value was associated with increased tailings utilisation and improved blended-aggregate gradation, which may contribute to fine-particle filling and microstructural densification tendencies [36]. RSM-based inverse design was then conducted over aggregate-to-cement ratios of 4–6 and slurry solids concentrations of 84–86%.

The response-surface equation predicted UCS for combinations of the aggregate-to-cement ratio and slurry solids concentration within the FCE-screened domain. Combinations were retained only when their predicted 28 d UCS exceeded 5 MPa. A slurry solids concentration of 84% was selected from the FCE-screened candidate range of 84–86% as the lower-bound value that satisfied the target strength requirement while maintaining favourable flowability for backfilling operations. This choice balanced strength and slurry workability [37,38]. Their strength and fresh-state performance were then experimentally examined to evaluate the consistency between the predicted and measured UCS values and the practical applicability of the proposed inverse-design approach (Table 8). The measured fresh-state properties further provided an assessment of the engineering suitability of the recommended mixtures.

The predicted 28 d UCS values of the three recommended schemes were 5.16, 6.96, and 9.12 MPa (Table 8). The corresponding measured values were 5.24, 6.85, and 9.21 MPa. The predictions closely matched the measurements, and all schemes satisfied the mine-strength requirement. As the aggregate-to-cement ratio decreased from 6 to 4, the design shifted from low cement consumption to high strength reserve. The slurries had slump values of 215–225 mm and bleeding rates of 3.24–3.51%, indicating good flowability and water-retention stability while meeting the strength requirement.

In summary, FCE narrowed the original design space to candidate ranges of the mix-design factors, while RSM provided continuous strength prediction and parameter inversion within those ranges. Their coupling provides a basis for target-strength-oriented mixture design of waste rock–unclassified tailings cemented backfill.

## 5. Conclusions

The main conclusions are as follows:

(1) The response-surface analysis quantitatively revealed the nonlinear relationship between mix-design parameters and 28 d UCS. Within the investigated domain, decreasing the aggregate-to-cement ratio and increasing the slurry solids concentration and unclassified tailings replacement ratio were associated with increased UCS. The factor influence followed the order of aggregate-to-cement ratio > slurry solids concentration > unclassified tailings replacement ratio, indicating that binder availability was the dominant factor affecting strength development.

(2) The interaction analysis demonstrated that the aggregate-to-cement ratio–slurry solids concentration interaction was the most significant among the investigated factor pairs. This interaction was associated with the combined effects of binder dosage and water-state regulation, resulting in different strength responses under varying factor combinations.

(3) The LF-NMR and SEM observations provided qualitative evidence for interpreting the strength variations among different mixtures. The observed changes in T_2_ distributions and microscopic morphologies suggested that variations in the unclassified tailings replacement ratio, aggregate-to-cement ratio, and slurry solids concentration were associated with changes in pore characteristics, hydration-product morphology, and particle-contact conditions. These observations were consistent with the measured strength trends and helped explain the possible microstructural mechanisms underlying the effects of mix-design factors.

(4) The coupled RSM–FCE inverse-design framework successfully transformed the target UCS requirement into candidate mix-design ranges and specific mixture schemes. The identified ranges (35–40% unclassified tailings replacement ratio, 4–6 aggregate-to-cement ratio, and 84–86% slurry solids concentration) were used to select three recommended mixtures, which were experimentally verified with measured UCS values of 5.24–9.21 MPa. These results support the feasibility and engineering applicability of the proposed target-strength-oriented design strategy for waste rock–unclassified tailings cemented backfill.

## Figures and Tables

**Table 1:** Basic physical properties of waste rock and unclassified tailings.

Material	Apparent Density (g/cm^3^)	Bulk Density (g/cm^3^)	Packing Density (%)	Porosity (%)	Moisture Content (%)
Waste rock	2.74	1.61	58.76	41.24	4.97
Unclassified tailings	2.68	1.58	58.96	41.04	15.24

**Table 2:** Chemical compositions of the raw materials.

Material	Chemical Composition (wt.%)							
	SiO_2_	Al_2_O_3_	Fe_2_O_3_	MgO	K_2_O	CaO	TiO_2_	Others
Waste rock	48.81	9.37	9.74	12.29	1.94	15.94	1.48	0.43
Unclassified tailings	71.69	12.65	8.89	2.88	1.28	0.99	0.44	1.18
Cement	23.49	7.82	2.52	1.83	1.13	58.05	0.42	4.74

**Table 3:** Response-surface factor levels.

Factor	Level		
	−1	0	1
Unclassified tailings replacement ratio (%)	25	32.5	40
Aggregate-to-cement ratio	4	7	10
Slurry solids concentration (%)	80	83	86

**Table 4:** Response-surface experimental results.

Run	Factor Levels (x_1_, x_2_, x_3_)			Uniaxial Compressive Strength (MPa)	
				28 d	
				Measured (Mean ± SD)	Predicted
1	0	0	0	2.62 ± 0.40	2.81
2	0	1	−1	0.91 ± 0.37	1.29
3	1	1	0	1.48 ± 0.31	1.31
4	0	−1	−1	5.48 ± 0.12	5.45
5	−1	0	1	5.07 ± 0.29	5.28
6	0	−1	1	11.89 ± 0.95	11.51
7	1	0	−1	2.57 ± 0.41	2.36
8	0	0	0	2.53 ± 0.15	2.81
9	0	0	0	2.98 ± 0.28	2.81
10	−1	1	0	1.07 ± 0.17	0.83
11	0	0	0	2.88 ± 0.41	2.81
12	1	0	1	5.27 ± 0.22	5.41
13	−1	−1	0	7.07 ± 0.29	7.24
14	0	1	1	2.43 ± 0.29	2.46
15	−1	0	−1	1.25 ± 0.10	1.11
16	0	0	0	3.05 ± 0.35	2.81
17	1	−1	0	7.88 ± 0.20	8.12

**Table 5:** Analysis of variance (ANOVA) for the quadratic response-surface model of 28 d UCS.

Source	Sum of Squares	df	Mean Square	F-Value	*p*-Value
Model	134.56	9	14.95	131.19	<0.0001
A–UTR	0.9385	1	0.9385	8.23	0.0240
B–A/C	87.32	1	87.32	766.18	<0.0001
C–SSC	26.10	1	26.10	229.02	<0.0001
AB	0.0400	1	0.0400	0.3510	0.5722
AC	0.3136	1	0.3136	2.75	0.1411
BC	5.98	1	5.98	52.45	0.0002
A^2^	0.0058	1	0.0058	0.0513	0.8273
B^2^	10.78	1	10.78	94.61	<0.0001
C^2^	2.47	1	2.47	21.64	0.0023
Residual	0.7978	7	0.1140	—	—
Lack of Fit	0.5919	3	0.1973	3.83	0.1136
Pure Error	0.2059	4	0.0515	—	—
Cor Total	135.35	16	—	—	—

Note: A, B, and C denote the unclassified tailings replacement ratio, aggregate-to-cement ratio, and slurry solids concentration, respectively. The em dash (—) indicates that the corresponding value is not applicable.

**Table 6:** Mixture designs and strengths used for grade classification.

Run	Unclassified Tailings Replacement Ratio (%)	Aggregate-to-Cement Ratio	Slurry Solids Concentration (%)	Uniaxial Compressive Strength (MPa)
				28 d
1	25	10	80	0.730
2	30	8	82	1.374
3	35	6	84	5.274
4	40	4	86	12.250

**Table 7:** Strength grades and corresponding factor ranges.

Strength Grade		Grade 1	Grade 2	Grade 3
Strength range (MPa)	Uniaxial compressive strength	0.730–1.374	1.374–5.274	5.274–12.250
Factor range	Unclassified tailings replacement ratio (%)	25–30	30–35	35–40
	Aggregate-to-cement ratio	10–8	8–6	6–4
	Slurry solids concentration (%)	80–82	82–84	84–86

**Table 8:** Recommended mixtures and verification results.

Scheme	Unclassified Tailings Replacement Ratio (%)	Aggregate-to-Cement Ratio	Slurry Solids Concentration (%)	Predicted 28 d UCS (MPa)	Measured 28 d UCS (MPa), Mean ± SD	SLUMP (mm), Mean ± SD	Bleeding Rate (%), Mean ± SD
Low-cement-consumption scheme	40	6	84	5.16	5.24 ± 0.18	215 ± 2.1	3.51 ± 0.28
Strength–economy balanced scheme	40	5	84	6.96	6.85 ± 0.22	220 ± 1.5	3.35 ± 0.24
High-strength-margin scheme	40	4	84	9.12	9.21 ± 0.41	225 ± 2.6	3.24 ± 0.21

## Data Availability

The original contributions presented in this study are included in the article. Further inquiries can be directed to the corresponding author.

## Nomenclature

**Symbol**	**Definition**
*d*_50_	Median particle size
*T*_2_	Transverse relaxation time in LF-NMR analysis
*y*	28 d uniaxial compressive strength (UCS), MPa
*x*_1_	Standardised coded variable for the unclassified tailings replacement ratio in the RSM model
*x*_2_	Standardised coded variable for the aggregate-to-cement ratio in the RSM model
*x*_3_	Standardised coded variable for the slurry solids concentration in the RSM model
*R* ^2^	Coefficient of determination
$R_{\mathrm{Adj}}^{2}$	Adjusted coefficient of determination
$R_{\mathrm{Pred}}^{2}$	Predicted coefficient of determination
PRESS	Predicted residual error sum of squares
*F*	F-statistic in analysis of variance
*p*	Probability value for statistical significance testing
*U*	Factor set in the FCE model
*u_i_*	*i*th factor in the FCE factor set
*u*_1_	Unclassified tailings replacement ratio
*u*_2_	Aggregate-to-cement ratio
*u*_3_	Slurry solids concentration
*V*	Evaluation set in the FCE model
*v_j_*	*j*th strength grade in the FCE set
*μ_ij_*(*u_i_*)	Membership function of factor *u_i_* with respect to strength grade *v_j_*
*β_i_*	Linear main-effect coefficient of the *i*th factor in the standardised RSM model
*w_i_*	Weight of the *i*th factor in the FCE model
*W*	FCE factor-weight vector
*W*_28*d*_	FCE factor-weight vector for the 28 d strength evaluation
*R*	Fuzzy relation matrix
*r_ij_*	Membership degree of the *i*th factor in the *j*th strength grade
*B*	Fuzzy comprehensive evaluation vector
*B_j_*	Overall membership degree of a mixture in the *j*th strength grade
*i*	Factor index, *i* = 1,2,3
*j*	Strength-grade index, *j* = 1,2,3
